# Research on Improved GPC of Pantograph Considering Actuator Time Delay and External Disturbance

**DOI:** 10.3390/s24227350

**Published:** 2024-11-18

**Authors:** Ying Wang, Yixuan Wang, Xiaoqiang Chen, Yuting Wang, Aiping Ma

**Affiliations:** 1 School of Automation and Electrical Engineering, Lanzhou Jiaotong University, Lanzhou 730070, China; wangying01@mail.lzjtu.cn (Y.W.); xqchen@mail.lzjtu.cn (X.C.); 12221546@stu.lzjtu.edu.cn (Y.W.); 2 Key Laboratory of Opto-Technology and Intelligent Control Ministry of Education, Lanzhou Jiaotong University, Lanzhou 730070, China; 3 Lanzhou High-Speed Railway Infrastructure Section, Lanzhou Railway Bureau, Lanzhou 730050, China; 15107065049@163.com

**Keywords:** pantograph, active control, improved generalized predictive control, time delay, external disturbance

## Abstract

Active control of pantograph is an effective method to improve the current received quality in electrified railway systems. To alleviate the negative impact of time delay in pantograph actuator, a Controlled Auto-Regressive Integrated Moving Average (CARIMA) model was designed for pantograph active control. In conjunction with the Euler-Bernoulli catenary model, an improved generalized predictive control (IGPC) algorithm was proposed, and its stability was analyzed. Then, the control performance was verified and discussed through testing. Subsequently, the effects of external disturbances and time delay on control performance were discussed. The results indicate that the proposed controller with a larger control gain, exhibits better performance in reducing fluctuation in contact force between pantograph and catenary, despite being affected by external disturbance and actuator time delay, it still shows significant control performance.

## 1. Introduction

**Figure 1:**
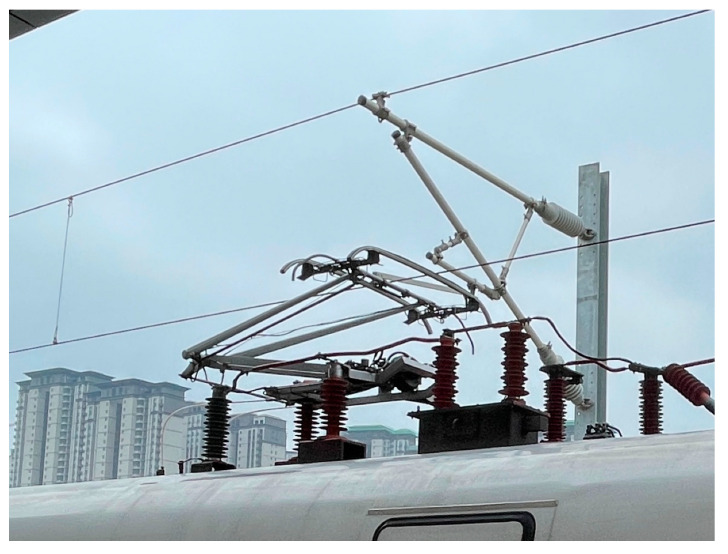
Pantograph-catenary system.

In modern high-speed railway systems [1,2], the dynamic behavior of the pantograph is easily influenced by various factors such as parameter matching and external random disturbances, due to the increase in train speed and changes in track conditions. These factors can result in instability of the contact force in the pantograph-catenary system, thereby increasing the risk of system failure. Therefore, understanding the dynamic relationship between the pantograph and catenary is crucial for ensuring the stability and safety of the train power supply. As an integral part of power transmission, the pantograph directly affects current collection quality and power supply reliability [3,4]. Incorporating active control into the pantograph can reduce contact force with the catenary, thus improving current collection quality. Previous research on active control has demonstrated that this approach not only enhances received current quality but also reduces fatigue on contact wires and wear on pantographs. It effectively minimizes fluctuations in contact force while being adaptable to different environmental conditions. However, precise control over pantograph motion remains a challenge due to complex random interference and actuator time delay within the system. The structure of Pantograph-Catenary System (PCS) discussed in this paper is illustrated in Figure 1. The pantograph is securely mounted on top through a supporting insulator while other components include: contact wire, dropper, messenger wire, support device, and related connecting parts [5,6,7].

Regarding the active control of pantograph, Song et al. studied the active control of contact force of the pantograph-catenary system in high-speed railways in terms of multi-body system modeling. By comparing the multi-body model and simulation results, they presented the key control methods for improving the dynamic stability of the system [8]. Wilk et al. proposed a new method for estimating the inertia and dissipation parameters of the railway pantograph model [9]. Some scholars have respectively proposed the optimal active control and optimal semi-active control methods of pantograph [10,11,12]. Shi et al. proposed an adaptive terminal sliding-mode control for pantograph based on optimal load [13]. Some scholars have proposed robust control algorithms for pantograph [14,15]. Hui et al. improved reinforcement learning through different methods to achieve fast-adaptive control of active pantograph in high-speed railways [8,16,17]. Regarding actuator time delay, Xie et al. studied the optimal control of high-speed railway pantograph considering actuator time delay [18]. Verriest et al. studied the structural properties of systems with vanishing time-variant delay based on the pantograph (scale-delay) equation [19]. Some scholars have considered time delay in the controller and discussed the corresponding control effect. Hajishafieiha et al. proposed a numerical solution for fractional-order pantograph delay differential equations [20]. Regarding external disturbances, existing research mainly focuses on external random disturbances such as wheel-rail irregularities, catenary irregularities, and other factors as well as the disturbance of external environmental wind. Some scholars have discussed the impact of the external wind environment on the pantograph-catenary system [21,22]. Duan et al. proposed a lumped-mass catenary model based on a disturbance observer for the design and verification of pantograph [23]. Pombo et al. studied the influence of environmental and track disturbances on the interaction between multiple pantograph and catenary in high-speed trains [24]. Yang et al. identified the short-wavelength contact wire irregularities of the pantograph-catenary system in electrified railways [25].

In recent years, generalized predictive control method has been widely used in the industrial field. Thanks to its predictive function, it can predict the system state in advance and calculate the control force in advance, thus having good control performance. However, traditional generalized predictive control requires the solution of multi-step Diophantine equations [26,27,28], considering the high-speed nature of electric locomotives, and controller latency issues need to be addressed, this paper proposes an improved generalized predictive controller for the pantograph [29,30]. This approach aims (1) to solve the actuator time-delay problem while increasing controller speed and (2) to consider the impact of external random disturbances on controller performance, focusing primarily on white noise and external environmental wind interference, thereby improving the real-time performance of the controller. We adopt improved generalized predictive control for pantographs to reduce the fluctuation of contact force, thereby improving the current collection quality of pantograph-catenary coupling and optimizing the pantograph-catenary system’s performance under high-speed railway conditions.

**Figure 2:**
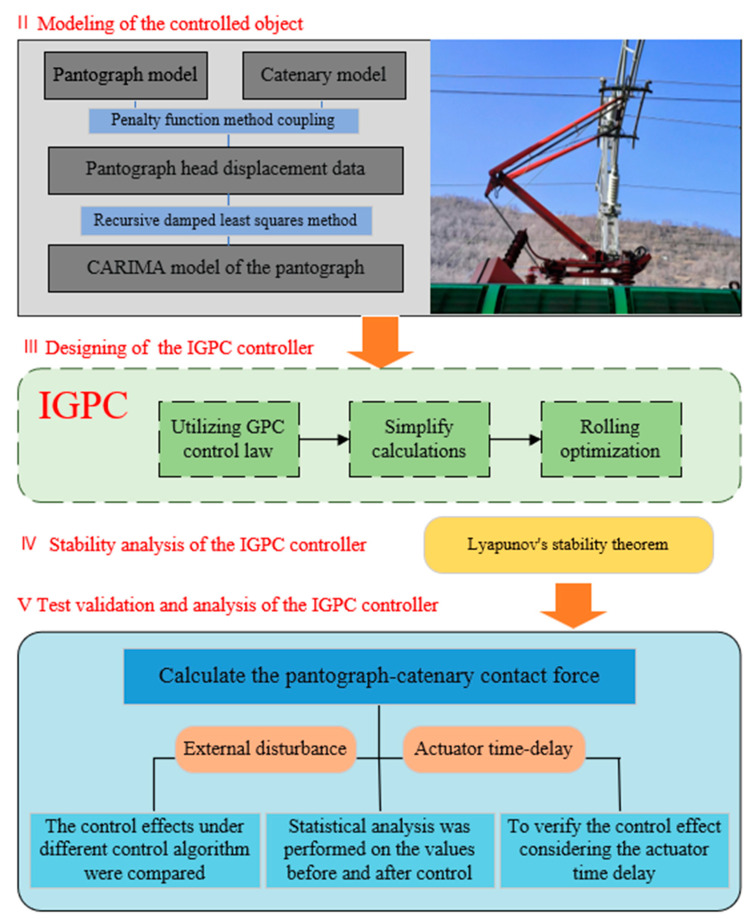
The overall framework and plan of this paper.

The main innovation points of this research are as follows: (a) The CARIMA model of the pantograph is established; (b) The GPC algorithm is improved; (c) The stability of IGPC is analyzed; (d) The influences of actuator time-delay and external disturbances are considered, and the control performance under different conditions is discussed. Figure 2 is the frame diagram of the paper. First, establish the controlled object model. The pantograph is reduced to a mass block model, and the catenary is equivalent to a two-segment Euler-Bernoulli beam model with constant tension. Then, the penalty function coupling is adopted. Next, an improved GPC controller is designed and the stability of the controller is verified by using the Lyapunov function. Then, the control performance of the proposed controller is tested and analyzed by adding random disturbances and time delay.

## 2. Modeling of the Controlled Object

### 2.1. Pantograph Modeling

The IGPC algorithm requires the derivation of control laws through the system’s CARIMA model, and the pantograph displacement parameters are identified using the least squares method to obtain the pantograph CARIMA model. In practical applications, only real-time displacement data from the pantograph is needed from sensors. However, due to the lack of a large amount of measured data for simulation analysis in the simulation process, it is necessary to establish a coupled PCS model. By simulating the displacement data of the pantograph from the established pantograph-catenary coupled model and performing recursive damped least squares parameter identification on the data, the CARIMA model of the pantograph is obtained.

**Figure 3:**
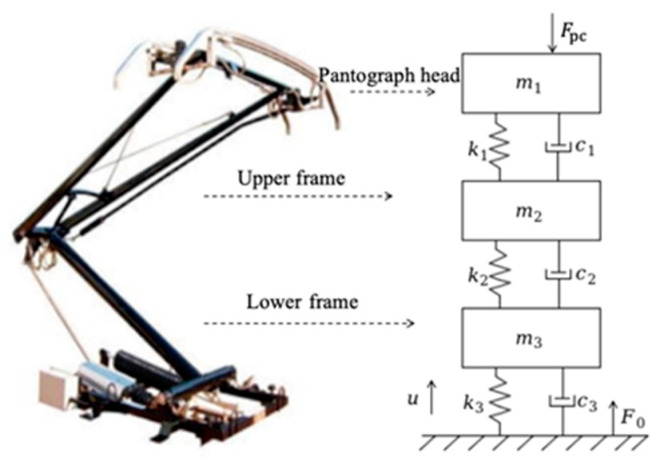
Pantograph lumped-mass model.

This paper establishes a pantograph lumped-mass model to accurately characterize the physical properties of the pantograph, as shown in Figure 3.

In Figure 3, *m*_1_, *m*_2_, *m*_3_ represent the equivalent masses of the pantograph head, upper frame, and lower frame respectively; *c*_1_, *c*_2_, *c*_3_ represent the equivalent damping coefficients of the pantograph head, upper frame, and lower frame respectively; *k*_1_, *k*_2_, *k*_3_ represent the equivalent spring stiffness between the pantograph head and the upper frame, between the upper and lower frames, and between the lower frame and the base respectively; *F*_pc_ represents the coupled contact force between the pantograph and the catenary; *u* represents the active control force of the pantograph; *F*_0_ represents the static uplift force of the pantograph on the rooftop. Based on multibody dynamics, the motion equations for the pantograph are derived [15].

(1) $$\left\{\begin{array}{l} m_{1}{\ddot{x}}_{1}+c_{1}({\dot{x}}_{1}-{\dot{x}}_{2})+k_{1}(x_{1}-x_{2})=F_{\mathrm{pc}}\\ m_{2}{\ddot{x}}_{2}+c_{1}({\dot{x}}_{2}-{\dot{x}}_{1})+c_{2}({\dot{x}}_{2}-{\dot{x}}_{3})+k_{1}(x_{2}-x_{1})+k_{2}(x_{2}-x_{3})=0\\ m_{3}{\ddot{x}}_{3}+c_{2}({\dot{x}}_{3}-{\dot{x}}_{2})+c_{3}{\dot{x}}_{3}+k_{2}(x_{3}-x_{2})+k_{3}x_{3}=F_{0}+u(t) \end{array}\right.$$

In the equations, *x*_1_, *x*_2_, and *x*_3_ represent the displacements of the pantograph head, upper frame, and lower frame respectively; ${\dot{x}}_{1}$, ${\dot{x}}_{2}$, ${\dot{x}}_{3}$ represent the velocities of the pantograph head, upper frame, and lower frame respectively; ${\ddot{x}}_{1}$, ${\ddot{x}}_{2}$, ${\ddot{x}}_{3}$ represent the accelerations of each mass block; *F*_pc_ is the dynamic contact force between the pantograph and the catenary; *F*_0_ is the static uplift force provided by the base to the pantograph; *u*(*t*) is the real-time control force. Summarizing and organizing Equation (1), the matrix form of the pantograph motion equation is obtained:

(2) $$M_{p}{\ddot{x}}_{p}+C_{p}{\dot{x}}_{p}+K_{p}x_{p}=F_{p}$$

where **M**_p_, **C**_p_, and **K**_p_ represent the equivalent mass matrix, equivalent damping matrix, and equivalent stiffness matrix, respectively. Among them:

$$M_{p}=\left[\begin{array}{ccc} m_{1} & 0 & 0\\ 0 & m_{2} & 0\\ 0 & 0 & m_{3} \end{array}\right],\;C_{p}=\left[\begin{array}{ccc} c_{1} & -c_{1} & 0\\ -c_{1} & c_{1}+c_{2} & -c_{2}\\ 0 & -c_{2} & c_{2}+c_{3} \end{array}\right],\;K_{p}=\left[\begin{array}{ccc} k_{1} & -k_{1} & 0\\ -k_{1} & k_{1}+k_{2} & -k_{2}\\ 0 & -k_{2} & k_{2}+k_{3} \end{array}\right],\;F_{p}=\left[\begin{array}{c} F_{\mathrm{pc}}\\ 0\\ F_{0}+u(t) \end{array}\right]$$

The pantograph model selected is DSA380, and its structural parameters are shown in Table 1:

### 2.2. Catenary Modeling

**Figure 4:**
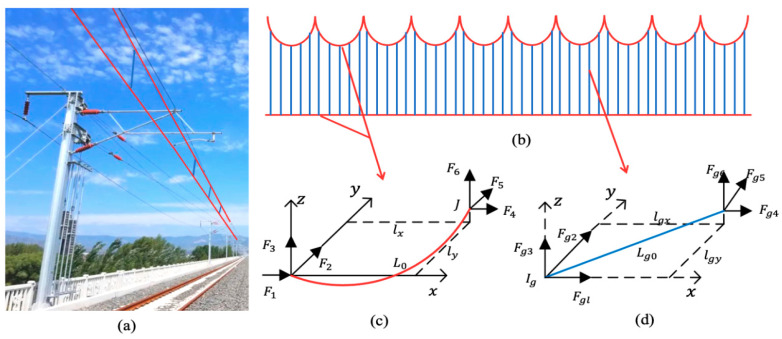
Nonlinear Model of the Catenary System: (**a**) Catenary system diagram (**b**) Catenary geometry (**c**) Nonlinear cable element (**d**) Nonlinear rod element.

The catenary system is an essential component of electrified railways, through which electric trains obtain electrical energy. The types of catenary systems are mainly divided into simple chain suspension, elastic chain suspension, and compound chain suspension. This paper focuses on the simple chain suspension, which is commonly used in China, with the modeling and research subject. Its specific parameters are shown in Table 2. Establishing a catenary simulation model, as shown in Figure 4:

The contact and sliding between the pantograph and the catenary constitute the key link in the normal operation of the PCS, involving complex physical phenomena and interactions. During the contact process, a specific contact force exists between the pantograph head and the catenary wire, and this pressure value is crucial for ensuring favorable electrical contact. For instance, an excessive contact force will exacerbate the wear of both the pantograph head and the catenary. When the contact force is too low, arcs are prone to be generated, thereby affecting the power-receiving quality. Meanwhile, the relative speed, surface roughness, and lubrication conditions between the two jointly influence the magnitude of friction and energy loss. The variations of these factors will not only impact the service life of the pantograph and the catenary but also exert a significant influence on the train’s current-receiving stability. Due to the high-speed running of the vehicle, the contact point between the pantograph and the catenary presents certain rigid characteristics. Meanwhile, different anchor sections of the catenary are fixed through compensation devices. This paper equates the messenger wire and the contact wire to two segments of Euler-Bernoulli beam models with constant tension. Since the vibration of the catenary is affected by multiple devices, conducting dynamic analysis on each part is complex. Therefore, simplifications are made to the simple chain suspension model during the modeling process. By introducing a lumped mass matrix **M**_c_, a damping matrix **C**_c_, and an overall stiffness matrix **K**_c_, the vibration equation of the catenary can be expressed in the following form [15]:

(3) $$M_{c}{\ddot{x}}_{c}+C_{c}{\dot{x}}_{c}+K_{c}x_{c}=F_{c}$$

where **M**_c_, **C**_c_, and **K**_c_ represent the mass, damping, and stiffness matrices respectively; ${\ddot{x}}_{c}$, ${\dot{x}}_{c}$, and $x_{c}$ denote the overall generalized acceleration, velocity, and displacement vectors respectively; **F**_c_ represents the lift force.

### 2.3. Pantograph—Catenary Coupled Modeling

**Figure 5:**
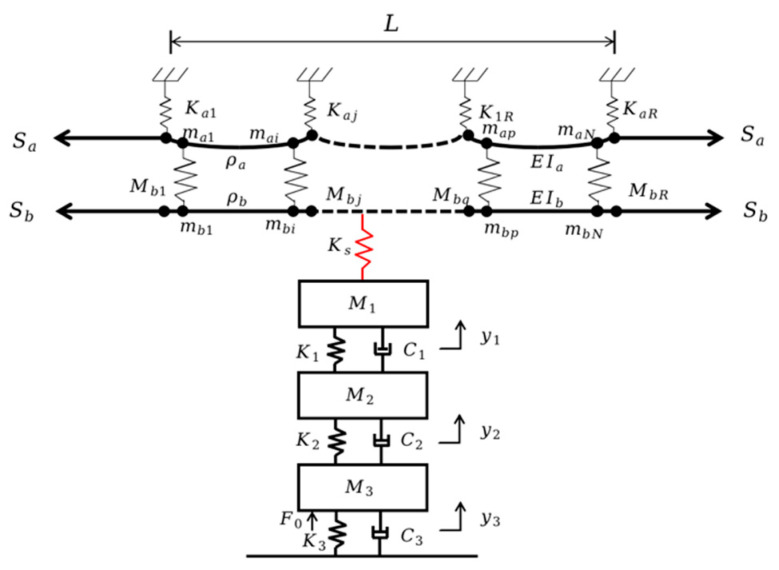
Pantograph-catenary coupling model.

In the above research, the pantograph model and catenary model have been established, and the dynamic behaviour of the coupling of the catenary system has been further analyzed to complete the establishment of the coupling model of the PCS. Figure 5 shows the schematic diagram of the coupling model of the catenary The pantograph adopts a traditional three-mass model. By coupling the pantograph model with the catenary model, the motion differential equation of the PCS when the pantograph moves on the contact element is obtained.

According to Equations (2) and (3), the pantograph-catenary coupling system model can be represented as:

(4) $$M_{\mathrm{pc}}{\ddot{X}}_{\mathrm{pc}}+C_{\mathrm{pc}}{\dot{X}}_{\mathrm{pc}}+K_{\mathrm{pc}}X_{\mathrm{pc}}=F$$

where,

$$\begin{array}{c} M_{\mathrm{pc}}=diag(M_{c},M_{p});\;C_{\mathrm{pc}}=diag(C_{c},C_{p})\\ {\ddot{X}}_{\mathrm{pc}}={\left[{\ddot{X}}_{c}\cdot {\ddot{X}}_{p}\right]}^{T};\;{\dot{X}}_{\mathrm{pc}}={\left[{\dot{X}}_{c}\cdot {\dot{X}}_{p}\right]}^{T};\;X_{\mathrm{pc}}={\left[X_{c}\cdot X_{p}\right]}^{T};\\ K_{\mathrm{pc}}=diag(K_{p},K_{c});\;F=diag(F_{p},F_{c}); \end{array}$$

### 2.4. Pantograph CARIMA Modeling

Establish a predictive pantograph CARIMA model. The controller constructed using the CARIMA model exhibits robustness to the dynamics of the modeled system. It naturally integrates the integral action into the control rate, thereby inherently eliminating deviations caused by disturbances. The mathematical model of the controlled object is as follows:

(5) $$A(z^{-1})y(k)=B(z^{-1})u(k-1)+C(z^{-1})V(k)/\Delta$$

Type, the output *y*(*k*) as the pantograph head displacement, the *u*(*k*) as the control input, {*V*(*k*)} as zero-mean noise sequence, *z*^−1^ as the backward-shift operator, as the $\Delta =1-z^{-1}$ for the difference operator, *A*(*z*^−1^), *B*(*z*^−1^) and *C*(*z*^−1^) are polynomials of the backward shift operator *z*^−1^, where:

(6) $$\left\{\begin{array}{c} \begin{array}{c} A(z^{-1})=1+a_{1}z^{-1}+\ldots +a_{n_{a}}z^{-n_{a}}\\ B(z^{-1})=b_{0}+b_{1}z^{-1}+\ldots +b_{n_{b}}z^{-n_{b}}\\ C(z^{-1})=1+c_{1}z^{-1}+\ldots +c_{n_{c}}z^{-n_{c}} \end{array} \end{array}\right.$$

For simplicity, *C*(*z*^−1^) = 1 is assumed, so that the model of the controlled object is simplified as follows.

(7) $$A(z^{-1})y(k)=z^{-d}B(z^{-1})u(k-1)+V(k)/\Delta$$

The CARIMA model can predict future system behaviour, which is necessary to achieve effective control. In the process of model parameter identification, the least square method is a common method, but the least square method also has some shortcomings. As the skew variance matrix decreases, the parameter explosion phenomenon is prone to occur. Adding the damping term of parameter variation to the objective function of parameter identification of the least squares method can increase the stability of the algorithm. Adding a damping term to the least squares method, the objective function of the recursive least squares method can be obtained as follows [31]:

(8) $$\left[{\left(Y_{m}-H_{m}{\hat{\theta }}_{m}\right)}^{T}W_{m}\left(Y_{m}-H_{m}{\hat{\theta }}_{m}\right)\right]+\mu {\left[{\hat{\theta }}_{m}-{\hat{\theta }}_{m-1}\right]}^{T}\left[{\hat{\theta }}_{m}-{\hat{\theta }}_{m-1}\right]$$

where, *µ*(*µ* > 0) is the damping factor, and the magnitude of *µ* describes the relative importance of the increment of the independent variable when the objective function *J* is taken to a pole. When *µ* = 0, the recursive damped least squares method becomes the general least squares method.

$$Y_{m}=H_{m}\theta_{m}+v_{m},Y_{m}=\left[\begin{array}{l} y(1)\\ \ldots \\ y(n) \end{array}\right]=\left[\begin{array}{l} Y_{m-1}\\ y_{m} \end{array}\right]\;H_{m}=\left[\begin{array}{cccccc} -y\left(0\right) & \cdots & -y\left(1-n\right) & u\left(0\right) & \cdots & u\left(1-n\right)\\ \vdots & \; & \vdots & \vdots & \; & \vdots \\ -y\left(m-1\right) & \cdots & -y\left(m-n\right) & u\left(m-1\right) & \cdots & u\left(m-n\right) \end{array}\right]=\left[\begin{array}{c} H_{m-1}\\ h_{m} \end{array}\right]$$

$$W_{m}=diag(\begin{array}{cccc} \lambda^{m-1} & \lambda^{m-2} & \cdots & \lambda^{0} \end{array})=\left(\begin{array}{cc} W_{m-1} & 0\\ 0 & 1 \end{array}\right)$$

$$V_{m}={\left[\begin{array}{ccc} v\left(1\right) & \cdots & v\left(m\right) \end{array}\right]}^{T}=\left[\begin{array}{c} V_{m-1}\\ v_{m} \end{array}\right]$$

According to the extreme value theorem, $\hat{\theta }$ should be satisfied for Equation (8) to hold

(9) $$\begin{array}{c} \frac{\partial J}{\partial \theta }{|}_{\theta =\hat{\theta }}=-2H_{m}^{T}W_{m}\left(Y_{m}-H_{m}{\hat{\theta }}_{m}\right)+2\mu \left[{\hat{\theta }}_{m}-{\hat{\theta }}_{m-1}\right]=0 \end{array}$$

The above equation can be sorted out

(10) $$\begin{array}{c} \left[\mu I+H_{m}^{T}W_{m}H_{m}\right]{\hat{\theta }}_{m}=\mu {\hat{\theta }}_{m-1}+H_{m}^{T}W_{m}Y_{m} \end{array}$$

Since $\mu I+H_{m}^{T}W_{m}H_{m}$ is invertible, there is a unique solution to Equation (10), which is

(11) $$\begin{array}{c} {\hat{\theta }}_{m}={\left[\mu I+H_{m}^{T}W_{m}H_{m}\right]}^{-1}\left[\mu {\hat{\theta }}_{m-1}+H_{m}^{T}W_{m}Y_{m}\right] \end{array}$$

let

(12) $$\begin{array}{c} P_{m-1}^{-1}=\mu I+H_{m-1}^{T}W_{m-1}H_{m-1} \end{array}$$

we can derive that

(13) $$\begin{array}{c} P_{m}={\left[\mu I+H_{m}^{T}W_{m}H_{m}\right]}^{-1} \end{array}$$

Upon substituting (13) into (11), it follows that…

(14) $$\begin{array}{c} {\hat{\theta }}_{m}=P_{m}\left[\mu {\hat{\theta }}_{m-1}+H_{m}^{T}W_{m}Y_{m}\right] \end{array}$$

thereby obtaining

(15) $$\begin{array}{c} H_{m-1}^{T}W_{m-1}H_{m-1}=P_{m-1}^{-1}{\hat{\theta }}_{m-1}-\mu {\hat{\theta }}_{m-2} \end{array}$$

By inserting Equations (5)–(7) into Equation (13), it follows that

(16) $$\begin{array}{c} P_{m}^{-1}=\mu I+\lambda H_{m-1}^{T}W_{m-1}H_{m-1}+h_{m}^{T}h_{m} \end{array}$$

Upon further simplifying Equation (16) by adding *μI* to each side, the result is

(17) $$\begin{array}{c} \mu I+H_{m}^{T}W_{m}H_{m}+\mu I=\mu I+\frac{1}{\lambda }\left[P_{m}^{-1}-\mu I-h_{m}^{T}h_{m}\right] \end{array}$$

Comparing Equation (12) with Equation (16), we find that

(18) $$\begin{array}{c} P_{m-1}^{-1}=\mu I+\frac{1}{\lambda }\left[P_{m}^{-1}-\mu I-h_{m}^{T}h_{m}\right] \end{array}$$

By inserting Equations (5)–(7) into Equation (14), it follows that

(19) $$\begin{array}{c} {\hat{\theta }}_{m}=P_{m}\left[\mu {\hat{\theta }}_{m-1}+\lambda H_{m-1}^{T}W_{m-1}y_{m-1}+h_{m}^{T}y_{m}\right] \end{array}$$

By inserting Equations (15)–(18) into Equation (19), it follows that

(20) $$\begin{array}{c} {\hat{\theta }}_{m}={\hat{\theta }}_{m-1}+\lambda \mu P_{m}\left[{\hat{\theta }}_{m-1}-{\hat{\theta }}_{m-2}\right]+P_{m}h_{m}^{T}\left[y_{m}-h_{m}{\hat{\theta }}_{m-1}\right] \end{array}$$

Combining Equations (19) and (20), the formula for the recursive damped least-squares method is derived as

(21) $${\hat{\theta }}_{m}={\hat{\theta }}_{m-1}+\lambda \mu P_{m}\left[{\hat{\theta }}_{m-1}-{\hat{\theta }}_{m-2}\right]+P_{m}h_{m}^{T}\left[y_{m}-h_{m}{\hat{\theta }}_{m-1}\right]\;\begin{array}{c} P_{m}={\left[\mu I+\lambda H_{m-1}^{T}W_{m-1}y_{m-1}+h_{m}^{T}h_{m}\right]}^{-1} \end{array}$$

In the context of the equation, ${\hat{\theta }}_{m-1}$ denotes the parameter estimate from the preceding time instance; $y_{m}-h_{m}\theta_{m-1}$ signifies the predictive error, which emanates from the discrepancy between the prior estimate ${\hat{\theta }}_{m-1}$ and the true parameters, this is also termed as innovation; $P_{m}h_{m}^{T}$ serves as the coefficient for rectifying the bias, known as the gain matrix; ${\hat{\theta }}_{m-1}-{\hat{\theta }}_{m-2}$ represents the divergence between the estimates at the last two moments; $\lambda ,\;\mu ,\;P_{m}$ are coefficients associated with the estimative discrepancies, encompassing elements such as the damping factor, the forgetting factor, and the covariance of the estimation error.

**Figure 6:**
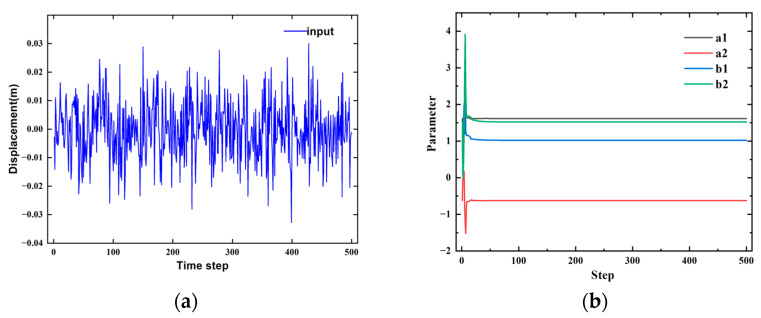
(**a**) Pantograph head displacement data (**b**) Results of parameter identification.

The recursive damped least square method is used for identification. The input parameters and identification results are shown in Figure 6, and the identified parameters are shown in Table 3.

From Figure 6, the identification values stabilize after 100 steps. For caution, the system iteration value at the 500th step is used to establish the CARIMA model for the pantograph:

(22) y(k)+1.61591y(k−1)−0.62445y(k−2)=1.02023u(k−1)+1.52059u(k−2)+V(k)

By minimizing the error inherent in the least squares method, we derive the parameters for the CARIMA model of the pantograph head displacement, thus ensuring the accuracy of the system model identification. The least squares method exhibits unbiasedness, efficiency, and consistency even under the perturbation of white noise, thereby avoiding extraneous interferences during open-loop step response experiments.

To evaluate the concordance between the identified model and the empirical data, the Theil inequality coefficient is selected as the criterion for evaluating the model’s performance, which is articulated as follows:

(23) $$TIC=\frac{\sqrt{\sum_{i}{\left(y_{i}-{\hat{y}}_{i}\right)}^{2}}}{\sqrt{\sum_{i}y_{i}^{2}}+\sqrt{\sum_{i}{\hat{y}}_{i}^{2}}}$$

In the specific case under examination, *y_i_* signifies the empirical measure of the pantograph head displacement, whereas symbolizes the predicted output derived from the recognition model. As stipulated in Equation (23), the Theil Inequality Coefficient (TIC) for the established model is 0.0402. This outcome underscores the robust congruence achieved by the model identified through the application of the recursive damped least squares technique, rendering it a viable instrument for further investigation within the realm of pantograph control research.

## 3. Designing of the IGPC Controller

**Figure 7:**
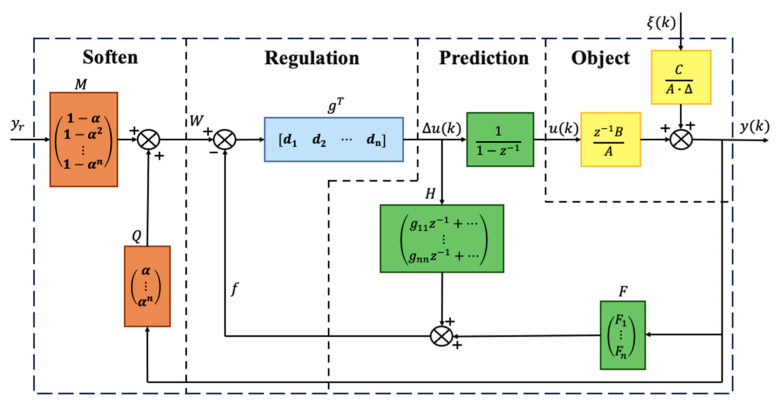
IGPC control diagram.

The GPC algorithm requires complex calculations and involves recursive solving of the Diophantine equation, as well as matrix inversion computations. Therefore, implementing the basic GPC algorithm demands considerable computational effort and time, significantly reducing the controller’s real-time performance. To address this issue, an IGPC algorithm is presented below. It allows for direct identification of controller parameters, eliminating the need for recursive solving of the Diophantine equation, thereby substantially reducing computation time. Figure 7 illustrates the structural diagram of the IGPC principle.

As shown in Figure 7, the main functions of IGPC are divided into four modules: softening, adjustment, prediction, and object. The roles of these four functions are briefly described as follows: The softening function makes the passive object smoothly approach the reference value, thereby avoiding overshoot. The adjustment function helps to finetune the parameters to achieve the desired control performance. The prediction function enables the controller to anticipate the system’s behavior and take proactive control actions. The object function defines the control objective and guides the controller to optimize the system’s performance. Among them, *y*_r_ is the reference value input, *y*(*k*) is the control output, and *ξ*(*k*) is the disturbance input.

The IGPC directly adopts the optimal control law from the GPC [30].

(24) $$\Delta U={\left(G^{T}G+\lambda I\right)}^{-1}G^{T}\left(W-f\right)$$

From Equation (24), it is evident that to calculate ∆*U*, it is necessary to separately determine the matrix *G* and the open-loop prediction vector *f*. As understood from GPC, there are *n* parallel predictive controllers:

(25) $$\left\{\begin{array}{c} y(k+1)=g_{0}\Delta u(k)+f(k+1)+E_{1}\xi (k+1)\\ y(k+2)=g_{1}\Delta u(k)+g_{0}\Delta u(k+1)+f(k+2)+E_{2}\xi (k+2)\\ \cdots \\ y(k+n)=g_{n-1}\Delta u(k)+\cdots +g_{0}\Delta u(k+n-1)+f(k+n)+E_{n}\xi (k+n) \end{array}\right.$$

From the last equation of Equation (25), it can obtain:

(26) $$y(k+n)=g_{n-1}\Delta u\left(k\right)+\ldots +g_{0}\Delta u(k+n-1)+f(k+n)+E_{n}\xi (k+n)$$

Let,

$$X(k)=[\Delta u(k),\Delta u(k+1),\ldots ,\Delta u(k+n-1),1],\theta (k)={\left[g_{n-1},g_{n-2},\cdots ,g_{0},f(k+n)\right]}^{T}$$

Equation (26) can be rewritten as:

(27) $$y(k+n)=X(k)\theta (k)+E_{n}\xi (k+n)$$

The predicted output value is:

(28) y(k+n|k)=X(k)θ(k)

and

(29) y(k|k+n)=X(k−n)θ(k)

At time instant *k*, if the elements of *X*(*k* − *n*) are known and $E_{n}\xi (k+n)$ is white noise, the parameter vector *θ*(*k*) can be estimated using ordinary least squares. However, $E_{n}\xi (k+n)$ is typically not white noise. Therefore, a method combining control strategy with parameter estimation is adopted, that is, using the estimated value of the auxiliary output prediction $\hat{y}(k|k-n)$ to replace the output prediction *y*(*k*|*k* − *n*), and considering the difference between $\hat{y}(k|k-n)$ and the actual value *y*(*k*) as white noise *ε*(*k*).

According to:

(30) $$\hat{y}(k|k-n)+\varepsilon (k)=y(k|k-n)+E_{n}\xi (k)\;y(k)-\hat{y}(k|k-n)=\varepsilon (k)$$

It can obtain that:

(31) $$y(k)=\hat{X}(k-n)\theta (k)+\varepsilon (k)$$

The parameter vector *θ*(*k*) can be estimated using the following recursive least squares formula:

(32) $$\hat{\theta }(k)=\hat{\theta }(k-1)+K(k)[y(k)-\hat{X}(k-1)\hat{\theta }(k-1)]\;K(k)=P(k-1){\hat{X}}^{T}(k-n){\left[\lambda_{1}+\hat{X}(k-n)P(k-1){\hat{X}}^{T}(k-n)\right]}^{-1}\;P(k)=\left[I-K(k)\hat{X}(k-n)\right]P(k-1)/\lambda_{1}$$

In the formula, *λ*_1_ is the forgetting factor, where 0 < *λ*_1_ < 1. Using the recursive formula above, the estimated value of *θ*(*k*), denoted as $\hat{\theta }$(*k*), can be obtained. From this, the elements *g*_0_, *g*_1_, …, *g*(*n* − 1) in matrix *G* and *f*(*k* + *n*) can be determined.

The *n*-step estimated value at time *k* can be calculated using the following equation:

(33) $$\hat{y}(k+n|k)=\hat{X}(k)\hat{\theta }(k)$$

where,

$$\hat{X}(k)=\left[\Delta u(k),\Delta u(k+1),\cdots ,\Delta u(k+n-1),1\right]$$

In the formula, ∆*u*(*k*), ∆*u*(*k* + 1), …, ∆*u*(*k* + *n* − 1) are replaced with the control increment calculated in the previous step at the corresponding point. The vector *f* in GPC is:

(34) $$\left[\begin{array}{c} y_{0}(k+1)\\ y_{0}(k+2)\\ \vdots \\ y_{0}(k+p-1)\\ y_{0}(k+p) \end{array}\right]=\left[\begin{array}{c} \hat{y}(k+2|k)\\ \hat{y}(k+3|k)\\ \vdots \\ \begin{array}{c} \hat{y}(k+p-1|k)\\ \hat{y}(k+p|k) \end{array} \end{array}\right]=\left[\begin{array}{c} h_{2}\\ h_{3}\\ \vdots \\ \begin{array}{c} h_{p-1}\\ h_{p} \end{array} \end{array}\right]e(k+1)$$

In the formula, *p* is the model time domain length (*p* ≥ *n*), *h*_2_, *h*_3_, …, *h*_p_ are error correction coefficients. *e*(*k* + 1) = *y*(*k* + 1)−*y*(*k* + 1|*k*) is the prediction error, where here we take *h*_2_ = *h*_3_ = … = *h*_p_ = 1. Due to the equivalence between *f* and *Y*_0_, using Equation (19), the next time step’s prediction vector *f* can be obtained:

(35) $$f=\left[\begin{array}{c} f(k+1)\\ f(k+2)\\ \vdots \\ f(k+p-1)\\ f(k+p) \end{array}\right]=\left[\begin{array}{c} \hat{y}(k+2|k)\\ \hat{y}(k+3|k)\\ \vdots \\ \hat{y}(k+p-1|k)\\ \hat{y}(k+p|k) \end{array}\right]+\left[\begin{array}{l} 1\\ 1\\ \vdots \\ 1\\ 1 \end{array}\right]e(k+1)$$

Once *G* and *f* are obtained, the control quantity can be calculated using Equation (11). At each step of the calculation, an *n*-step control sequence is obtained for this and subsequent steps. To make timely use of feedback information to determine the control quantity, only the first control quantity in the sequence is applied to the system at each instance, while the remaining *n*−1 control quantities are not directly applied but are used solely for the calculation of $\hat{Y}$.

In the self-tuning algorithm, as seen from Equation (11), each calculation must solve an *n* × *n* dimensional inverse matrix ${\left(G^{T}G+\lambda I\right)}^{-1}$ online. Here, same as GPC, a control horizon length *m* (*m* ≤ *n*) is introduced. When *j* > *m*, ∆*u*(*k* + *j* − *i*) = 0, thereby reducing matrix *G* to *n* × *m* dimensions, and the matrix $\left(G^{T}G+\lambda I\right)$ becomes an *m* × *m* square matrix, decreasing the dimensionality and computational workload, thus significantly saving computation time. The essence of the IGPC is to find the optimal control increment sequence ∆*U* that minimizes the objective function value.

## 4. Stability Analysis of the IGPC Controller

Using the CARIMA model mentioned above to describe the controlled object, multiplying both sides of Equation (24) by the difference operator ∆ gives [32]:

(36) $$\begin{array}{c} A(z^{-1})\Delta y(k)=B(z^{-1})\Delta u(k-1)+C(z^{-1})\xi (k) \end{array}$$

From the above equation, it can obtain that:

(37) $$\begin{array}{l} Y\left(k+1\right)=\left[\begin{array}{ccccc} -a_{1} & -a_{2} & \cdots & -a_{n} & -a_{n+1}\\ 1 & 0 & \cdots & 0 & 0\\ \vdots & \vdots & \ddots & \vdots & \vdots \\ 0 & 0 & \cdots & 1 & 0 \end{array}\right]\left[\begin{array}{c} y\left(k\right)\\ y\left(k-1\right)\\ \vdots \\ 0 \end{array}\right]+\\ \left[\begin{array}{cccc} b_{0} & b_{1} & \cdots & b_{m}\\ 0 & 0 & \cdots & 0\\ \vdots & \vdots & \ddots & \vdots \\ 0 & 0 & 0 & 0 \end{array}\right]\left[\begin{array}{c} \Delta u\left(k\right)\\ \Delta u\left(k-1\right)\\ \vdots \\ \Delta u\left(k-m\right) \end{array}\right]=AY\left(k\right)+BU\left(k\right) \end{array}$$

Taking the state variables as:

(38) $$\begin{array}{c} X\left(k\right)={\left[\begin{array}{cccccc} y\left(k\right) & \cdots & y\left(k-n\right) & \Delta u\left(k-1\right) & \cdots & \Delta u\left(k-m\right) \end{array}\right]}^{T} \end{array}$$

The state equations of the controlled object are as follows:

(39) $$\begin{array}{c} X\left(k+1\right)=\psi X\left(k\right)+\Gamma \Delta u\left(k\right) \end{array}$$

where,

$$\psi =\left[\begin{array}{cccccccccc} -a_{1} & -a_{2} & \cdots & -a_{n} & -a_{n+1} & b_{0} & b_{1} & \cdots & b_{m-1} & b_{m}\\ 1 & 0 & \cdots & 0 & 0 & & & & & \\ \vdots & 1 & & & 0 & & & & & \\ & & & \vdots & \vdots & & & 0_{n\times m} & & \\ 0 & & & 1 & 0 & & & & & \\ & & & & & 0 & 0 & \cdots & 0 & 0\\ & & & & & 1 & 0 & \cdots & 0 & 0\\ & & 0_{m\times \left(n+1\right)} & & & 0 & 1 & \cdots & 0 & 0\\ & & & & & \vdots & \vdots & & \vdots & \vdots \\ & & & & & 0 & 0 & \cdots & 1 & 0 \end{array}\right]$$

$$\Gamma =\left[\begin{array}{ccccccccccc} b_{0} & 0 & 0 & \cdots & 0 & \vdots & 1 & 0 & 0 & \cdots & 0 \end{array}\right]$$

let $X^{\prime }\left(k\right)=\left[\begin{array}{c} X\left(k+1\right)\\ X\left(k\right) \end{array}\right]$ it obtain that:

(40) $$\begin{array}{c} X^{\prime }\left(k+1\right)=\left[\begin{array}{cc} \psi & 0\\ I & 0 \end{array}\right]\left[\begin{array}{c} X\left(k\right)\\ X\left(k-1\right) \end{array}\right]+\left[\begin{array}{c} \Gamma \\ 0 \end{array}\right]\Delta u\left(k\right) \end{array}$$

From Equation (24), it follows that:

(41) $$\Delta U=\Phi G^{T}\left(\Delta \omega \left(k\right)-\Delta f\left(k\right)\right)=\;\Phi^{\prime }\left[\omega \left(k\right)-FY_{0}\left(k\right)-H\Delta U_{0}\left(k\right)\right]+\Phi^{\prime \prime }\left[\omega \left(k-1\right)-FY_{0}\left(k-1\right)-H\Delta U_{0}\left(k-1\right)\right]$$

where,

$$\Phi ={\left(\lambda I+G^{T}G\right)}^{-1},\Phi^{\prime }=\Phi G^{T},\Phi^{\prime \prime }=-\Phi G^{T},$$

$$Y_{0}\left(k\right)={\left[y\left(k\right),\cdots ,y\left(k-n\right)\right]}^{T},\Delta U_{0}\left(k\right)={\left[\Delta U\left(k\right),\cdots ,\Delta U\left(k-m\right)\right]}^{T}$$

$$F=\left[\begin{array}{cccc} f_{0}^{1} & f_{1}^{1} & \cdots & f_{n}^{1}\\ f_{0}^{2} & f_{1}^{2} & \cdots & f_{n}^{2}\\ \vdots & \vdots & \ddots & \vdots \\ f_{0}^{N} & f_{1}^{N} & \cdots & f_{n}^{n} \end{array}\right]$$

Then, at time *k*, the predictive control sequence is as follows:

(42) $$\begin{array}{c} \Delta u\left(k|k\right)=\Phi_{1}^{\prime }\left[w\left(k\right)-FY_{0}\left(k\right)-H\Delta U_{0}\left(k\right)\right]+\Phi_{1}^{\prime \prime }\left[w\left(k-1\right)-FY_{0}\left(k-1\right)-H\Delta U_{0}\left(k-1\right)\right]\\ \Delta u\left(k+1|k\right)=\Phi_{2}^{\prime }\left[w\left(k\right)-FY_{0}\left(k\right)-H\Delta U_{0}\left(k\right)\right]+\Phi_{2}^{\prime \prime }\left[w\left(k-1\right)-FY_{0}\left(k-1\right)-H\Delta U_{0}\left(k-1\right)\right]\\ \vdots \\ \Delta u\left(k+N-1|k\right)=\Phi_{N}^{\prime }\left[w\left(k\right)-FY_{0}\left(k\right)-H\Delta U_{0}\left(k\right)\right]\begin{array}{c} +\Phi_{N}^{\prime \prime }\left[w\left(k-1\right)-FY_{0}\left(k-1\right)-H\Delta U_{0}\left(k-1\right)\right] \end{array} \end{array}$$

At time *k* + 1, the predictive control sequence is as follows:

(43) $$\begin{array}{c} \Delta u\left(k|k+1\right)=\Phi_{1}^{\prime }\left[w\left(k+1\right)-FY_{0}\left(k+1\right)-H\Delta U_{0}\left(k+1\right)\right]+\Phi_{1}^{\prime \prime }\left[w\left(k\right)-FY_{0}\left(k\right)-H\Delta U_{0}\left(k\right)\right]\\ \Delta u\left(k+1|k+1\right)=\Phi_{2}^{\prime }\left[w\left(k+1\right)-FY_{0}\left(k+1\right)-H\Delta U_{0}\left(k+1\right)\right]+\Phi_{2}^{\prime \prime }\left[w\left(k\right)-FY_{0}\left(k\right)-H\Delta U_{0}\left(k\right)\right]\\ \vdots \\ \Delta u\left(k+N-1|k+1\right)=\Phi_{N}^{\prime }\left[w\left(k+1\right)-FY_{0}\left(k+1\right)-H\Delta U_{0}\left(k+1\right)\right]\begin{array}{c} +\Phi_{N}^{\prime \prime }\left[w\left(k\right)-FY_{0}\left(k\right)-H\Delta U_{0}\left(k\right)\right] \end{array} \end{array}$$

In the formula, $\Phi_{1}^{\prime }$ and $\Phi_{1}^{\prime \prime }$ are the *i*-th rows of $\Phi^{\prime }$ and $\Phi^{\prime \prime }$ respectively. At time *k*, the future control inputs are represented as:

(44) $$\Delta u\left(k|k\right)=u\left(k-1|k-1\right)+\Delta u(k|k)\;\Delta u\left(k+1|k\right)=u\left(k-1|k-1\right)+\Delta u\left(k|k\right)+\Delta u(k+1|k)\;\vdots \;\Delta u\left(k+N-1|k\right)=u\left(k-1|k-1\right)+\Delta u\left(k|k\right)+\overset{N-1}{\underset{i=1}{\sum }}\Delta u\left(k+i|k\right)$$

If there is a delay of *n* sampling periods at time *k* + 1, then it follows that:

(45) $$\Delta u(k+n|k+1)=u(k+n|k+1)-\Delta u(k+n|k)=\sum_{i=0}^{n}\Delta u(k+i|k+1)+\sum_{i=1}^{n}\Delta u(k+i|k)$$

From Equations (43)–(45), it can obtain that:

(46) $$\begin{array}{l} \Delta u\left(k+n|k+1\right)=\overset{n}{\underset{i=0}{\sum }}\{\Phi_{i+1}^{\prime }\left[w\left(k+1\right)-FY_{0}\left(k+1\right)-H\Delta U_{0}\left(k+1\right)\right]\\ +\Phi_{i+1}^{\prime \prime }\left[w\left(k\right)-FY_{0}\left(k\right)-H\Delta U_{0}\left(k\right)\right]\}-\overset{n}{\underset{i=0}{\sum }}\{\Phi_{i+1}^{\prime }\left[w\left(k\right)-FY_{0}\left(k\right)-H\Delta U_{0}\left(k\right)\right]\\ +\Phi_{i+1}^{\prime \prime }\left[w\left(k-1\right)-FY_{0}\left(k-1\right)-H\Delta U_{0}\left(k-1\right)\right]\} \end{array}$$

Combining with the state variables, the above equation is transformed into:

(47) $$\begin{array}{l} \Delta u\left(k+n|k+1\right)=\overset{n}{\underset{i=0}{\sum }}\Phi_{i+1}^{\prime }w\left(k+1\right)-\left[\overset{n}{\underset{i=0}{\sum }}\Phi_{i+1}^{\prime }F\overset{n}{\underset{i=0}{\sum }}\Phi_{i+1}^{\prime }H\right]X\left(k+1\right)\\ +\overset{n}{\underset{i=0}{\sum }}\Phi_{i+1}^{\prime \prime }w\left(k\right)-\left[\overset{n}{\underset{i=0}{\sum }}\Phi_{i+1}^{\prime \prime }F\overset{n}{\underset{i=0}{\sum }}\Phi_{i+1}^{\prime \prime }H\right]X\left(k\right)-\overset{n}{\underset{i=1}{\sum }}\Phi_{i+1}^{\prime }w\left(k\right)\\ +\left[\overset{n}{\underset{i=1}{\sum }}\Phi_{i+1}^{\prime }F\overset{n}{\underset{i=1}{\sum }}\Phi_{i+1}^{\prime }H\right]X\left(k\right)-\overset{n}{\underset{i=1}{\sum }}\begin{array}{c} \Phi_{i+1}^{\prime \prime }w\left(k-1\right)+\left[\overset{n}{\underset{i=1}{\sum }}\Phi_{i+1}^{\prime \prime }F\overset{n}{\underset{i=1}{\sum }}\Phi_{i+1}^{\prime \prime }H\right]X\left(k-1\right) \end{array} \end{array}$$

From Equation (44), it is known that:

(48) $$\Delta u(k|k)=u(k)=\Phi^{\prime }w(k)+\Phi^{\prime \prime }w(k-1)+[(-\Phi_{1}^{\prime }F-\Phi_{1}^{\prime }H)(\Phi_{1}^{\prime }F-\Phi_{1}^{\prime \prime }H)]X^{\prime }(k)$$

Since the system’s given values do not affect the stability analysis of the system, we set *w*(*k* − 1), *w*(*k*), and *w*(*k* + 1) to zero. Then, combining Equations (46)–(48), we have:

(49) $$\begin{array}{c} \Delta u\left(k+n|k+1\right)=-LX^{\prime }\left(k\right) \end{array}$$

where,

(50) $$\begin{array}{l} L=\left[\begin{array}{cc} \psi & 0\\ I & 0 \end{array}\right][\sum_{i=0}^{n}\Phi_{i+1}^{\prime }F\sum_{i=0}^{n}\Phi_{i+1}^{\prime }H\sum_{i=0}^{n}\Phi_{i+1}^{\prime \prime }F\sum_{i=0}^{n}\Phi_{i+1}^{\prime \prime }H]+\\ \left[\begin{array}{l} \Gamma \\ 0 \end{array}\right][-\Phi_{1}^{\prime }F-\Phi_{1}^{\prime }H-\Phi_{1}^{\prime \prime }F-\Phi_{1}^{\prime \prime }H]-[\sum_{i=1}^{n}\Phi_{i+1}^{\prime }F\sum_{i=1}^{n}\Phi_{i+1}^{\prime }H\sum_{i=1}^{n}\Phi_{i+1}^{\prime \prime }F\sum_{i=1}^{n}\Phi_{i+1}^{\prime \prime }H] \end{array}$$

Equation (49) can be used as the state feedback for Equation (37), and the state equation of the closed-loop system is:

(51) $$\begin{array}{c} X^{\prime }\left(k+1\right)=\left(\left[\begin{array}{cc} \psi & 0\\ I & 0 \end{array}\right]-\left[\begin{array}{c} \Gamma \\ 0 \end{array}\right]L\right)X^{\prime }\left(k\right) \end{array}$$

**Theorem** **1.** *In networked control systems with stochastic experiments, for any given positive definite symmetric matrix Q, if there exists a positive definite real symmetric matrix P that satisfies the following equation:*

(52) $$\begin{array}{c} {\left(\left[\begin{array}{cc} \psi & 0\\ I & 0 \end{array}\right]-\left[\begin{array}{c} \Gamma \\ 0 \end{array}\right]L\right)}^{T}P\left(\left[\begin{array}{cc} \psi & 0\\ I & 0 \end{array}\right]-\left[\begin{array}{c} \Gamma \\ 0 \end{array}\right]L\right)-P=-Q \end{array}$$

*Then the closed-loop system is asymptotically stable.*

**Proof.**  Let *V*(*k*) = *X*(*k*)*PX*(*k*), then:

(53) $$\begin{array}{l} \Delta V=V\left(k+1\right)-V\left(k\right)=X^{T}\left(k+1\right)PX\left(k+1\right)-X^{T}\left(k\right)PX\left(k\right)PX\left(k\right)\\ =-X^{T}\left(k\right)QX\left(k\right) \end{array}$$

According to Lyapunov’s stability theorem, if *Q* is positive definite, then ∆*V* is negative definite. This indicates that the closed-loop system of the proposed IGPC controller is asymptotically stable. □

## 5. Test Validation and Analysis of the IGPC Controller

### 5.1. Evaluation of Control Performance Considering External Disturbance

**Figure 8:**
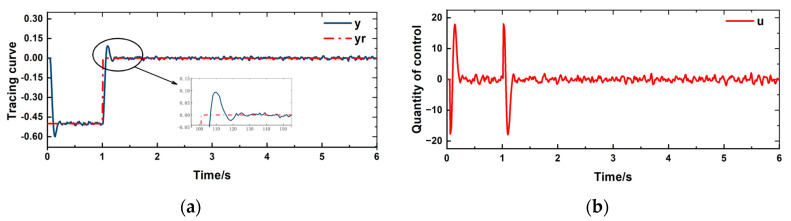
(**a**) Tracing curve (**b**) Control quantity change curve.

The given reference value *y*_r_ for controlling the displacement of the pantograph head is a square wave step signal from −0.5 to 0. The control objective is to reduce the vertical fluctuation of the pantograph head as much as possible. During the operation of the pantograph, the working conditions are complex and variable. Random external disturbances have been added to the simulation process to replicate the actual operating conditions of the pantograph. The control effect is shown in Figure 8a, an overshoot phenomenon occurs when the controlled quantity fits the tracking curve. The control quantity is shown in Figure 8b apart from the large fluctuation in the control quantity that occurs when there are significant changes in the tracking curve, there is no chattering phenomenon at other times.

Through deriving the CARIMA model of the pantograph and applying the IGPC algorithm for optimization, the specific parameters of its control algorithm are shown in Table 4.

**Figure 9:**
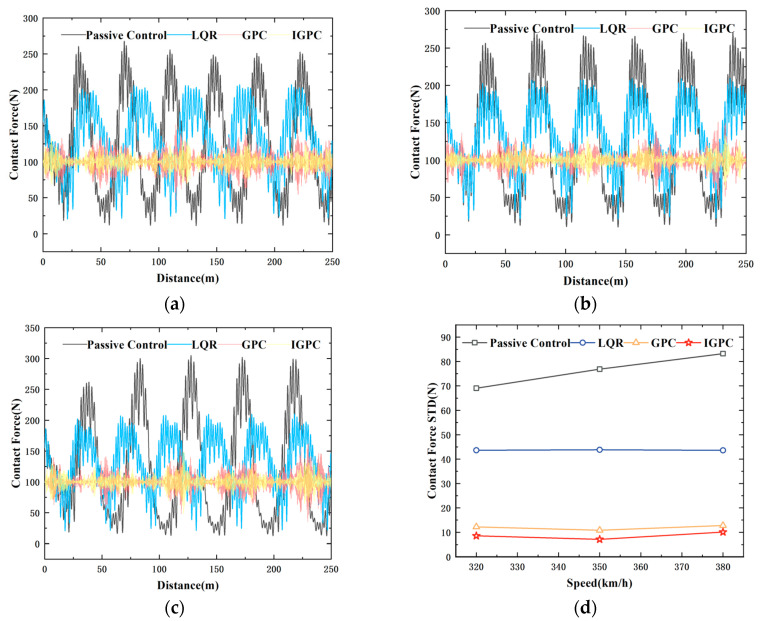
Contact force fluctuation comparison diagram (**a**) 320 km/h (**b**) 350 km/h (**c**) 380 km/h (**d**) Comparison chart of contact force standard deviation.

Considering that the maximum operating speed designed for the pantograph model established in this paper is 380 km/h, the scenarios simulated in this paper belong to the real-world operating speed. Figure 9 shows the comparative analysis of contact forces when passive control, LQR control, GPC, and IGPC operate over a distance of 250 m at speeds of 320 km/h, 350 km/h, and 380 km/h.

**Figure 10:**
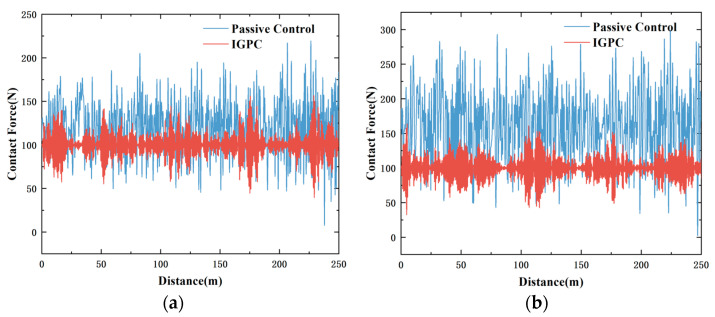
Contact force fluctuation diagram considering wind disturbance (**a**) 320 km/h (**b**) 380 km/h.

The diagram illustrates that both GPC and IGPC can effectively suppress fluctuations in contact force, reduce the standard deviation of contact force, and maintain the average contact force at approximately 100 N, thereby enhancing the quality of the current received. Then, 10 m/s cross wind was added to the system as a disturbance, and the control effect before and after IGPC control was compared. Figure 10 shows the simulation results of contact force comparison at 320 km/h and 380 km/h speed.

As can be seen from the simulation results, after considering the crosswind disturbance, the fluctuation of contact pressure under passive control increases significantly. After adding IGPC control, the fluctuation of contact pressure can be effectively reduced, indicating that this control method has strong robustness.

### 5.2. Evaluation of Control Performance Considering Controller Time Delay

When the time delay of actuator is 10 ms, the CARIMA model of the pantograph is as follows:

(54) y(k)+1.61591y(k−1)−0.62445y(k−2)=1.02023u(k−1)+1.52059u(k−2)+V(k)

When the time delay of the actuator is 20 ms, the CARIMA model of pantograph is as follows:

(55) y(k)+1.61591y(k−1)−0.62445y(k−2)=1.02023u(k−2)+1.52059u(k−3)+V(k)

The reference value *y*_r_ for controlling pantograph head displacement is a square wave step signal ranging from −0.5 to 0.

**Figure 11:**
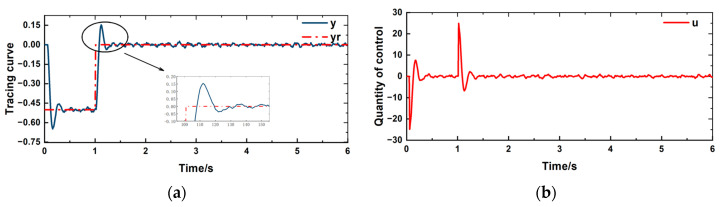
(**a**) Tracing curve (**b**) Control quantity change curve.

Figure 11 illustrates that the tracking capability remains effective; however, there is a significant increase in overshoot. Once the system stabilizes, there are no substantial fluctuation, indicating good control performance. It is evident that the proposed controller effectively addresses the time delay issue of the pantograph.

As a large and complex controlled object, the pantograph encounters numerous uncertainties during operation. Changes in operating conditions can lead to deviations between the identified model parameters and the actual parameters. Nevertheless, due to its rapid online identification speed, the functions of rolling optimization and feedback correction allow real-time control of the controlled object even under complex conditions. The issue of actuator time delay can also be adequately resolved. The simulation results reveal that the controller can still achieve effective control performance under external disturbances and when considering different time delay.

**Figure 12:**
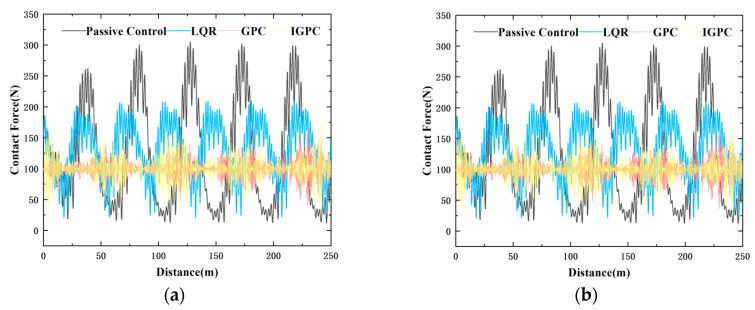
Contact Force with different control methods: (**a**) 10 ms time delay (**b**) 20 ms time delay.

As can be seen from Figure 10 above, regardless of whether the actuator time delay is considered, IGPC can still obtain relatively good control performance. This method can effectively solve the problem of reduced control effect caused by the time delay of the pantograph actuator. The simulation verification compares passive control and LQR control to verify the effectiveness of IGPC when the time delay is 10 ms and 20 ms respectively at a speed of 380 km/h, as shown in Figure 12.

Under the consideration of a 10 ms time delay, at speeds of 320 km/h, 350 km/h, and 380 km/h, the standard deviations of contact force under GPC and IGPC control are reduced by 82.31%, 84.12%, 85.91%,88.06% and 84.66%, 84.87% respectively compared to passive control. and 20ms time delay, at speeds of 320 km/h, 350 km/h, and 380 km/h, the standard deviations of contact force under GPC and IGPC control are reduced by 81.38%, 84.12%, 82.22%, 84.89%, and 76.92%, 80.68% respectively compared to passive control. As shown in Figure 12, with the increase in vehicle speed, the fluctuation of contact force intensifies. However, the simulation results show that at different speeds, both GPC and IGPC can significantly reduce the fluctuation of contact force and reduce the standard deviation of contact force. However the simulation time of GPC is 2 min and 5 s, and the simulation time of IGPC is 46 s. Under the condition that the control effects are both significant, the control speed of IGPC is greatly improved, effectively improving the current receiving quality of the pantograph and enhancing the real-time performance of control.

## 6. Discussion

In this study, we have proposed an enhanced GPC for the pantograph system that takes into account actuator time delay and external disturbances. The results obtained hold significant implications in the field of pantograph-catenary interaction control. The key discovery of this research lies in the successful design of the improved GPC controller, which effectively mitigates the adverse effects of time delay and external disturbances on the PCS. Under the guidance of this controller, the pantograph demonstrates improved stability and superior tracking capability compared to conventional control methods. For instance, the contact force between the pantograph and catenary remains within a narrower and more stable range, thereby reducing disconnection and wear risks—directly impacting reliable electric train operation. The enhancement in GPC controller performance can be attributed to meticulous consideration of actuator time delay and external disturbances during its design process. By incorporating appropriate compensation mechanisms, the controller can adapt to these challenging conditions. Stability analysis based on Lyapunov function provides a solid theoretical basis for understanding why the controller can maintain stability under such complex circumstances. Furthermore, analysis of results under random disturbances and time delay conditions demonstrates robustness of the controller—exemplified by its ability to withstand sudden external disruptions without compromising performance.

Previous studies on pantograph control mainly focused on idealized models, without fully considering real condition challenges such as actuator time delay and complex external disturbances [11]. Some studies proposed basic GPC controllers, but they often lacked the ability to effectively handle these non-ideal conditions. For example, ref. [30] only considered the nominal system without taking into account the possible variations and disturbances during actual operation.

This research fills the gap by introducing an improved GPC specifically designed to address these issues. The novelty of this approach lies in the combination of advanced control techniques to handle time delay and disturbance rejection simultaneously. This not only improves the control performance but also broadens the application scope of GPC in the PCS. Compared with existing methods, our controller provides a more practical and reliable solution for maintaining stable contact between the pantograph and the catenary under various operating conditions.

Description of Research Limitations: Despite the successful implementation of the improved GPC controller, our study has several limitations. Firstly, the model of the PCS may still be a simplification of the actual complex physical structure. There may be some unmodeled dynamics that could potentially affect the control performance under more extreme conditions. Secondly, the experimental validation was conducted under a set of predefined wind disturbance and time delay scenarios. In real-condition applications, the types and magnitudes of disturbances can be much more diverse.

Future research can focus on refining the pantograph-catenary model to include more detailed physical characteristics. This may involve more accurate representations of the mechanical flexibility of the pantograph structure and the complex behavior of the catenary under different weather and operating conditions. In addition, more extensive experimental studies with a wider range of disturbance scenarios should be carried out to further validate and improve the controller’s performance. Moreover, exploring the potential of integrating our controller with other advanced sensing and diagnostic technologies can provide a more comprehensive solution for pantograph-catenary interaction management, enabling real-time monitoring and adaptive control to ensure the highest level of operational reliability.

## 7. Conclusions

The main conclusions drawn from the research presented in this paper can be summarized as follows:

(1) Establishing the CARIMA model of the pantograph: A CARIMA model is proposed for the pantograph based on input/output data, providing a reliable basis for designing effective control strategies. contact and slide of pantograph and catenary.
(2) Improving the GPC algorithm: The traditional GPC algorithm is improved to reduce the calculation time and thereby enhance the control efficiency.
(3) Analyzed the stability of IGPC: The proposed IGPC controller is proved to be stable through the derivation of Lyapunov stability analysis.
(4) Considering the influence of actuator time delay and external disturbance: Through tests and verifications, the influences of actuator time delay and external disturbances were respectively discussed, and the suppression of the contact force fluctuation under different time delays by the controller was analyzed.

## Figures and Tables

**Table 1:** Basic Structural Parameters of DSA380 Type Pantograph.

Quality	Parameter Value (kg)	Damping	Parameter Value (Ns/m)	Stiffness	Parameter Value (N/m)
*m*_1_	6	*c*_1_	0	*k*_1_	4600
*m*_2_	7.12	*c*_2_	0	*k*_2_	14,100
*m*_3_	5.8	*c*_3_	70	*k*_3_	80

**Table 2:** Parameters of simple chain type catenary system.

Different Parameters	Specific Value
Tension of the bearing cable *S*_a_ (kN)	21
Tension of the catenary *S*_b_ (kN)	27
Elastic modulus of the bearing cable *EI*_a_ (N·m^2^)	310
Elastic modulus of the catenary *EI*_b_ (N·m^2^)	400
Span (m)	48
Number of strings per span	5
Dropper intervals (m)	5/9.5

**Table 3:** Identification parameters for the recursive damped least squares method.

Parameter	a1	a2	b1	b2
Estimated value (Iterate 50 times)	1.61654	−0.62509	1.02586	1.52707
Estimated value (Iterate 250 times)	1.61580	−0.62427	1.02035	1.52073
Estimated value (Iterate 500 times)	1.61591	−0.62445	1.02023	1.52059

**Table 4:** IGPC Controller parameters.

Algorithm Control Parameters	Numerical Value
Prediction length *N*_1_	8
Control length *N*_u_	4
Control weighting coefficient *λ*	0.8
Softening coefficient α	0.75

## Data Availability

Data are contained within the article.
